# H1N1 infection-induced thyroid storm

**DOI:** 10.4103/1817-1737.62475

**Published:** 2010

**Authors:** Salim Alawi Baharoon

**Affiliations:** *Intensive Care Departments, King Saud Bin Abdulaziz, University for Health Sciences, Saudi Arabia*

**Keywords:** Influenza A, H1N1, thyroid storm, infection induced thyroid dysfunction

## Abstract

A thyroid storm is a life-threatening exacerbation of thyrotoxicosis, and is usually characterized by hyperthermia, tachycardia, severe agitation and altered mental status. A thyroid storm may be triggered by many causes, including systemic pulmonary infections. Delay in prompt diagnosis leads to high mortality. We present the first case of H1N1 infection triggering a thyroid storm. The delay in diagnosis because of preoccupancy with the H1N1 pandemic may have contributed to the poor outcome. When assessing cases with H1N1 infection, physicians should be more vigilant in order not to miss other important diagnoses.

A thyroid storm is defined as a life-threatening exacerbation of thyrotoxicosis, and is usually characterized by hyperthermia, tachycardia, severe agitation and altered mental status.[1–3] It is a clinical emergency that, without early diagnosis and aggressive treatment, has a mortality rate as high as 20 to 30%.[1,2] The storm is usually due to a severe exacerbation of a preexisting thyrotoxicosis that leads to the failure of different organ systems, resulting in death.[4,5]

Infections are frequently cited as a precipitant of thyroid storms in patients with thyrotoxicosis.[6,7] In this case report, we present a case of thyroid storm presenting initially as H1N1 infection.

## Case Report

A 31-year-old Afghani female, living in Makkah Al Mukarrama was brought by her relatives during the Hajj season in 2009 to King Abdulaziz Hospital with a three-day history of high-grade fever, vomiting, cough, expectoration and increasing shortness of breath. The family denied previous history of any illnesses, though they had lately noticed that she always felt hot. No further information was available.

On examination in the emergency department, she was conscious but irritable. Recorded vitals in were as follows: temperature 39°C, HR 150 BPM, BP 100/55 mmHg and SaO_2_ 98% with 4 L simple face oxygen mask. Chest examination revealed presence of few bilateral basal crackles, while neurological examination showed normal reactive pupils with no lateralization signs or neck stiffness. There was no documentation of neck stiffness on initial assessment.

Laboratory results were as follows: white blood cell count (WBC) 37.7 × 10^9^/L (normal range [NR], 5.0–10.0 × 10^9^/L); hemoglobin, 12.0 g/dL (NR, 12.0–16.0 g/dL); serum glutamic oxaloacetic transaminase (AST) 33 U/L (NR, 10–35 U/L); serum glutamic pyruvic transaminase (ALT) 23 U/L (NR, 0–40 U/L); blood urea nitrogen, 12.5 mg/dL (NR, 8–20 mg/dL); creatinine, 0.9 mg/dL (NR, 0.6–1.5 mg/dL); blood sugar, 430 mg/dL (NR, 50–110 mg/dL); serum sodium, 140.9 mEq/L and urine dipstick glucose +++ Ketones ++.

Her chest X-ray showed bilateral interstitial infiltrate. Lactic acid measurement test was not available. Blood, sputum and urine cultures were all obtained. Nasopharyngeal swab for H1N1 was also obtained.

The patient was diagnosed with diabetic ketoacidosis and community-acquired bronchopneumonia with possible influenza A (H1N1) viral pneumonia. She was admitted to the medical ward and was started on Ceftriaxone, Clindamycine and Oseltamivir (Tamiflu). She also received insulin infusion and was given IV hydration with normal saline (NS).

After 48 h of being in the medical ward, the medical team decided to intubate the patient because of tachypnea, hypoxia and restlessness. Her arterial blood gas (ABG) at that time was as follows: PH 7.2 PaO_2_ 42 mmHg, pCO_2_ 17.7 mmHg, HCO_3_ 10.7 mEq/L. She remained in the medical ward for another 12 h until a bed could be arranged in the ICU.

In the ICU, the first set of vitals in ICU was as follows: temperature 39°C, HR 160 and BP 95/48 mm Hg. She was ventilated on Synchronized Intermittent Mandatory Ventilation (SIMV) mode with 60% FiO2, a tidal volume (V_t_) of 450 and positive end-expiratory pressure (PEEP) of 5 mm Hg. On that setting, the pulse oximetry was showing a saturation of (SaO_2_) 100%. A right subclavian central line was inserted and central venous pressure (CVP) monitoring was started. Her initial CVP was 8 mm Hg, she was given 6–8 L of IV fluid boluses in the first 12-h of care in the ICU and a maintenance fluid of 150–200 cc/h of NS. Inotropic support was started with norepinephrine, which was titrated to maintain systolic BP above 90 mm Hg. She was sedated with midazolam and fentanyl. She was started on piperacillin/tazobactam, and ciprofloxacin, while Tamiflu was continued. Her H1N1 PCR was reported positive within 6 h of admission to the unit.

She remained critically ill, and after 48 h, she was still febrile with a high-grade temperature of 39.5°C despite around the clock on paracetamol. She also had sinus tachycardia with a heart rate of 130–160. Her blood pressure (BP) was fluctuating on high-dose norepinephrine, which was changed to phenylephrine and vasopressin. All cultures including sputum and blood did not grow any organisms, and despite that antibiotic was changed again in 48 h to imipenem and ciprofloxacin because of poor response to the initial regimen.

Her FiO_2_ requirement, however was progressively improving and in 48 h, she was on 40% FiO_2_, with ABG pH 7.4 PaO_2_ 108 mm Hg, pCO_2_ 28 mm Hg and HCO_3_ 20 mEq/L. The bilateral chest x-ray infiltrate was resolving.

On the third day of admission, a thyroid function test was requested due to unexplained tachycardia. Thyroid function results were available in 24 h and were as follows Thyroid stimulating hormone TSH < 0.01 mIU/L (0.3–3.0 nmolIU/L), T3 50 nmol/L (1.1–3 nmol/L) T4 200 nmol/L (60–145 nmol/L).

The diagnosis of thyroid storm was considered and she was started on propylthiouracil, 150 mg every 6 h (the only available drug) and on esmolol infusion. No iodine preparation was available.

There was a significant improvement of tachycardia and hemodynamics after these measures but the patient was already progressing to multiorgan failure with progressively worsening renal and liver parameters as shown in Table 1. She was started on renal replacement therapy on the sixth day of admission. On the seventh day of admission, she suddenly became bradycardic and had a 6-min cardiac arrest. The patient's pupils were fixed and dilated after cardiopulmonary resuscitation. A CT of the brain was requested; it revealed a massive subarachnoid hemorrhage with severe brain edema. Despite aggressive measures that included Mannitol and hyperventilation, she progressed to brain death and was pronounced dead on the ninth day of admission to the hospital.

**Table 1:** Laboratory values during admission

				ICU Admission				Treatment of crisis				
												
	Day 1	Day 2	Day 3	Day 4	Day 5	Day 5	Day 6	Day 6	Day 7	Day 7	Day 8	Day 9
Urea mg/dL	68	24	66	39	52	74	95	127	130	134	196	240
Creatinine mg/dL	0.8	0.8	0.6	0.8	1.1	1.8	1.7	1.9	2.7	2.3	2.8	3.1
Glucose mg/dL	452	253	145	205	333	336	285	333	199	253	383	365
Na mEq/L	118	153	145	153	155	152	154	156	156	155	150	146
K mEq/L	5.3	3.4	2.8	2.2	3.9	4.6	4.2	2.7	2.7	3.3	5.4	5.3
CL mEq/L	92	116	103	102	118	116	121	118	117	116	116	110
CK U/L	439	87	ND	ND	ND	ND	ND	ND	78	ND	ND	ND
LDH U/L	268	ND	ND	ND	ND	ND	ND	ND	911	ND	ND	ND
AST U/L	61	ND	ND	ND	ND	ND	ND	ND	514	ND	ND	ND
ALT U/L	51	ND	ND	ND	ND	ND	ND	ND	120	ND	ND	ND
WBC ×10^9^/L	37.7	23	10	11	21	ND	16	ND	11	ND	15	14
Hb g/L	12	20	10	12	10	ND	9	ND	8.6	ND	9	9.7
PLT ×10^9^/L	309	111	108	136	190	ND	170	ND	102	ND	100	90
aPTT s	43	ND	ND	ND	42	ND	ND	ND	49	ND	ND	ND
INR	1.5	ND	ND	ND	1.34	ND	ND	ND	2.6	ND	ND	ND

ND: Not done

## Discussion

For a long time, researchers have been interested in infection as a factor in the pathogenesis of thyroid illness. Valtonen *et al.* measured a broad spectrum of bacterial and viral antibodies in paired sera of 32 patients with thyroid disease of recent onset including subacute thyroiditis, Graves' disease and Hashimoto's disease and found evidence of a preceding infection in 44% of the patients. Preceding bacterial infections were more common than viral infections in those samples.[7]

Infections are frequently cited as a potential precipitating factor of thyroid storms. Direct involvement of the thyroid gland (suppurative thyroiditis) with acute or subacute infection can precipitate a crisis.[8] However, the mechanism for precipitating a crisis in systemic illness is not well understood. It is well documented that the stress of systemic infections such as respiratory infections, endocarditis and urinary tract infections can precipitate a crisis.[9–13] Some researchers found acceleration of thyroxine and triiodothyronine turnover during systemic infection; this finding may partially explain the thyroid dysfunction during systemic illness,[14] Wolf *et al.* studied the sera of patient recovering from *Yersinia enterocolitica* infections and observed that the immunoglobulins of these patients exhibited Graves' disease-like activity in human thyroid membranes.[15]

Infections of viruses, including the hepatitis C, hepatitis B and Epstein–Barr virus infection were associated with increased incidence of clinical and subclinical autoimmune thyroiditis, which may represent an Immunmodulation phenomenon.[16,17] Alteration of thyroid hormones has also been observed in HIV infection especially at advanced stages.[18,19]

While the parainfluenza virus has previously been associated with one case of thyroid storm, neither Influenza A infection nor H1N1 strain infections has been previously cited as a precipitant for such a crisis. The fear of an H1N1 infection pandemic that occurred through out 2009 has caused much confusion, sometimes leading to delay in the diagnosis of some important illnesses and to misclassification of patient under the umbrella of H1N1 infection. The diagnosis of thyroid storm was delayed in our case because of the emphasis on H1N1 infection.

This case highlights the very important fact that physicians should be very vigilant when evaluating cases of H1N1 infection and should consider other diagnoses. Unfortunately, our patient progressed to multi-organ failure, which is a well-known complication of thyroid storm. To our knowledge, subarachnoid hemorrhage has not been reported as a complication of thyroid storm. Transient disturbances in consciousness level, central sinus venous thrombosis and bilateral ganglia infarct all have all been reported previously.[20–22] A distinct entity of high-attenuation areas appearing in the CT scans of patients after cardiac arrest (called pseudo-subarachnoid hemorrhage) has been reported in literature; this may be the finding in our patient. This type of hemorrhage is seen in post-cardiac arrest patients and is associated with severe brain edema and poor outcome.[23]

## Conclusion

This is the first case of H1N1 induced thyrotoxicosis that we are aware of. The case presented here illustrates how the obsession with H1N1 infection can potentially delay the diagnosis of other critical illnesses and adversely affect the outcome.
